# Mitochondrial Energy-Regulating Effect of Atractyloside Inhibits Hepatocellular Steatosis Through the Activation of Autophagy

**DOI:** 10.3389/fphar.2020.575695

**Published:** 2020-09-30

**Authors:** Pengfei Zhang, Lijun Li, Huimin Sun, Yipeng Zhang, Guoliang Zhang, Tianyu Zhang, Changchun Zeng

**Affiliations:** ^1^ Department of Medical Laboratory, Shenzhen Longhua District Central Hospital, Shenzhen, China; ^2^ State Key Laboratory of Respiratory Disease, Guangzhou Institutes of Biomedicine and Health, Chinese Academy of Sciences, Guangzhou, China; ^3^ University of Chinese Academy of Sciences, Beijing, China; ^4^ Department of Quality Control, Shenzhen Longhua District Central Hospital, Shenzhen, China; ^5^ Clinical Laboratory, Shenzhen Longhua District Central Hospital, Shenzhen, China; ^6^ National Clinical Research Center for Infectious Diseases, Guangdong Key Laboratory for Emerging Infectious Diseases, Shenzhen Third People’s Hospital, Southern University of Science and Technology, Shenzhen, China

**Keywords:** atractyloside, autophagy, hepatocellular steatosis, mitochondrial adaptation, triglyceride

## Abstract

**Background and Aim:**

Atractyloside (ATR), a mitochondrial uncoupler, is known for its specific inhibition of mitochondrial oxidative phosphorylation. Previous studies have reported that moderate mitochondrial uncoupling effect is beneficial to increase the decomposition and clearance of hepatic lipid, prevent the occurrence of fatty liver diseases. Moreover, the beneficial effects of mitochondrial uncouplers on type 2 diabetes and metabolic syndromes have been consistently observed. The present study investigated the effect of ATR on steatosis level of HepG2 cells treated with free fatty acid (FFA).

**Methods:**

Intracellular triglyceride level and Oil Red O staining were assessed, the mitochondrial adaptation and ADP/ATP ratio were analyzed, the protein level of AMPK, mTOR and LC3B, autophagic flux, and the co-localization of LC3B with lipid droplets was performed.

**Results:**

ATR treatment inhibited the activity of mitochondrial respiratory chain complexes I and IV, decreased the mitochondrial membrane potential, and increased the ADP/ATP ratio in the FFA-treated cells. Furthermore, ATR increased the gene expression and protein level of LC3B and promoted the autophagic flux processing from early autophagosome to late autolysosome by increasing the protein level of AMPKα and decreasing the protein level of mTOR. An increased number of autophagosomes (LC3B) was also observed in the lipid droplets. ATR treatment accelerated lipid degradation in the FFA-treated cells, and the lowest lipid content was observed in the cell group with 7.5 μM ATR.

**Conclusion:**

Low concentrations (2.5, 5, and 7.5 μM) of ATR treatment could activate autophagy to accelerate the degradation of TGs in steatosis HepG2 cells; the mechanism may be related to the activation of the AMPK/mTOR pathway induced by the increased ADP/ATP ratio. In addition, the ideal concentration of ATR for improving steatotic HepG2 cells was 7.5 μM.

## Introduction

Fatty liver disease (FLD) induced by the accumulation of excessive lipids in hepatocytes may lead to steatohepatitis and even cirrhosis accompanied with obesity, hyperglycemia, and other metabolic syndromes (Neuschwander-Tetri, 2017). The traditional treatments for FLD are diet control, moderate exercise, and hypolipidemic medical treatment, but most of them achieve limited success, and drugs specifically approved for FLD are unavailable (Neuschwander-Tetri, 2017). Several new pharmacological strategies act broadly to alter energy balance or specifically inhibit key enzymes involved in lipid synthesis (Samuel and Shulman, 2018). In addition, a novel class of liver-targeted mitochondrial uncoupling effects has been proven to increase hepatocellular energy expenditure and reverse metabolic and hepatic steatosis (Demine et al., 2019). The common characteristics of these new pharmacological treatments include ATP deprivation, activation of the energy-sensing enzyme of cells (i.e., adenosine 5-monophosphate activated protein kinase or AMPK) (Smith et al., 2016), and increased level of autophagy (Zhang X. et al., 2018). During energy deprivation, cellular lipids stored as triglycerides in lipid droplets (LDs) are engulfed by autophagosomes and delivered to lysosomes for degradation and use as an energy source (Singh et al., 2009). Loss of autophagy is associated with the accumulation of LDs upon exposure to a high concentration of free fatty acid (FFA). Furthermore, pharmacological up-regulation of autophagy reduces hepatotoxicity and steatosis in alcohol or non-alcohol fatty liver mouse models (Wang, 2015). Thus, pharmacological treatment targeting mitochondrial energy expenditure to induce a moderate level of autophagy may be advantageous for preventing or reversing hepatic steatosis and the associated metabolic complications.

**Figure:**
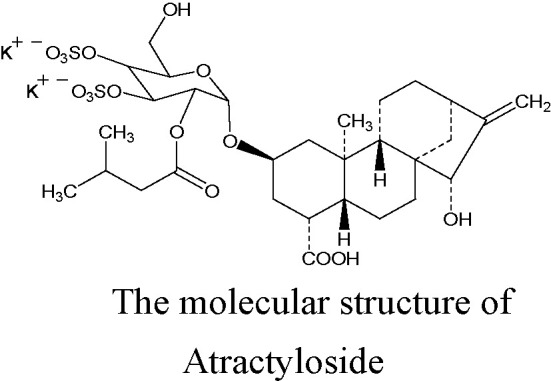


Atractyloside (ATR), a diterpenoid glycoside with one isovaleric and two sulfate groups, is mainly extracted from the fruits of *Xanthium Sibiricum*. Its molecular structure is shown in the figure (Kedrow et al., 2010). ATR is a high-affinity specific inhibitor of the mitochondrial ADP/ATP carrier, and it can induce a mitochondrial uncoupling effect. ATR competitively combines with adenine nucleotide transposase (ANT), inhibits the transport of ADP across the mitochondrial membrane, prevents the synthesis of ATP, and leads to a failure in energy balance; however, the inhibitory effect can be reversed by a high concentration of ADP (more than 200 μM) (Stewart and Steenkamp, 2000). A previous study demonstrated that ATR can induce the opening of the mitochondrial permeability transition pore (mPTP), leading to a reduction in ATP content in arteriolar smooth muscle cells (Song et al., 2012). Altered cellular energy expenditure is a key feature that has been reported to increase lipolysis, and decrease lipogenesis, the total triacylglycerol and circulating levels, and the triggering mechanism may be related to the activation of the AMPK signaling pathway (Demine et al., 2019). Moreover, systematic or liver-specific ANT2 cKO mice are lean and resistant to hepatic steatosis, obesity, and insulin resistance under a high fat diet, and the protection effect against fatty liver is partially through the pharmacologic inhibition of ANT2 by low-concentration carboxyatractyloside (Lee et al., 2014; Cho et al., 2017; Seo et al., 2019). These previous studies found that ATR has a potential to improve the steatosis of hepatocytes.

To prove this finding, the present study investigated the effect of ATR on the mitochondrial adaptation, AMPK-mTOR (mammalian target of rapamycin) signaling pathway, autophagy, and steatosis level of HepG2 cells treated with FFA. The results showed that ATR could increase the ADP/ATP ratio, activate the autophagy of cells, and accelerate the degradation of triglycerides in steatotic HepG2 cells. This work provides a theoretical basis for ATR-induced autophagy for the prevention and treatment of FLD.

## Methods

### Reagents

ATR (SA9830, 97% pure), oleic acid (OA, SC9320), and palmitic acid (PA, H8780) were purchased from Solarbio Life Science (Beijing China). HepG2 cells (SCSP-510) were purchased from American Type Culture Collection (ATCC, CL-173TM; Manassas, VA). L-02 cells (CL-0111) were purchased from Procell Life Science & Technology Co., Ltd (Wuhan China). Oil red O was purchased from Sigma-Aldrich (O0625; St. Louis, MO). Triglyceride (TG) assay kits was bought from Jiancheng Bioengineering Institute (Nanjing, China). AMPKα (5831), phospho-AMPKα (Thr 172, 2535), mTOR (2983), phospho-mTOR (Ser 2448, 2974), microtubule-associated protein 1 light chain 3B (LC3B, 3868), goat anti-rabbit IgG antibody combined with horseradish peroxidases (7074), and anti-rabbit IgG (H+L) (DyLight™ 680 conjugate; 5366) were purchased from Cell Signaling Technology, Inc. (Boston, MA). An ECL detection kit (Pierce™ ECL Western Blotting Substrate, Thermo Scientific, USA) was also used.

### FFA Preparation

OA and PA were prepared according to a previous method but with some modification (Gómez-Lechón et al., 2007). In brief, OA was dissolved in a 100 mmol/L (mM) NaOH solution at 70°C and mixed with 10% FFA bovine serum albumin (BSA) at 55°C. PA was dissolved in 200 mM NaOH at 70°C and mixed with 10% FFA BSA at 55°C. The 1 mM FFA stock solution was prepared by mixing OA and PA at a volume ratio of 2:1.

### Cell Culture

The HepG2 cells/L-02 cells were cultured in a 4.5 g/L glucose DMEM medium/1640 medium with 10% (v/v) fetal bovine serum (FBS) and 1% penicillin/streptomycin solution (100 units/ml penicillin and 100 μg/ml streptomycin) in an atmosphere of 5% CO_2_ and 95% humidified air at 37°C.

### Cell Viability Assay

The cells were seeded on a 96-well plate (5 × 10^3^ cells per well) and cultured until they adhered stably. Then, the cells were treated with 0, 2.5, 5, 7.5, 10, and 20 μM ATR for 48 h. Cell viability was assessed using cell counting kit-8 (CCK-8, Meilun Biotechnology, Dalian, China) in accordance with the manufacturer’s instruction. In brief, the culture medium was removed after 48 h of incubation with ATR, and 10 μl of CCK-8 solution with 90 μl of the culture medium was added to each well. Then, the cells were incubated in 95% humidified air and 5% CO_2_ for 2 h at 37°C. The absorbance was measured at 450 nm by using a microplate reader (Bio-Rad, Hercules, CA).

### ADP/ATP Ratio Assay

For this assay, cells were seeded on a white opaque 96-well plate (5 × 10^3^ cells per well) and treated with 0, 2.5, 5, 7.5, 10, and 20 μM ATR. The ADP/ATP ratio was determined at 3, 6, 12, 24, and 48 h. An assay based on a luciferin-luciferase reaction was performed using the Abnova ADP/ATP Assay Kit (Walnut, CA, USA) in accordance with the manufacturer’s protocol. The assay involved two steps. First, cells were lysed using the working reagent to release ATP and ADP. In the presence of luciferase, ATP immediately reacted with the substrate D-luciferin to produce light. The light intensity was a direct measure of the intracellular ATP concentration. Second, ADP was enzymatically converted to ATP, which reacted with D-luciferin as in the first step.

### HepG2 Cells Steatosis and ATR Treatment

HepG2 cells were seeded on two six-well plates (6 × 10^5^ cells per well). After reaching 80% confluence, the cells were treated with and without 1 mM FFA (mixture of OA/PA at a ratio of 2:1) for 24 h. Then, the cells that had been exposed to FFA were treated with ATR at different concentrations (0, 2.5, 5, 7.5, and 10 μM) for 24 h.

### Oil Red O Staining and Measurement of Intracellular TG Level

For oil red O staining, the cells were washed with sterile phosphate buffer saline (PBS) and fixed with 4% paraformaldehyde in PBS for 10 min at room temperature. Subsequently, the cells were washed thrice with PBS, incubated with 60% isopropanol for 15 s, and stained with freshly prepared oil red O solution for 10 min. Afterward, the cells were washed thrice with distilled water and imaged under an inverted light microscope (Axio Vert. A1, Carl Zeiss, Germany). Meanwhile, oil red O was extracted with 100% isopropanol, and the absorbance was measured at 510 nm to obtain quantitative results.

For the measurement of cellular triglyceride (TG) content, cells were harvested, washed with cold PBS, homogenized in ice-cold PBS under ultrasonic fragmentation (power: 20%; ON time: 5 s; OFF time: 10 s; repeats: 15), and centrifuged at 1346×g for 10 min at room temperature to obtain the supernatant. The TG level in each sample was estimated using a commercially available kit purchased from Nanjing Jiancheng Bioengineering Institute (Nanjing, Jiangsu, China) in accordance with the manufacturer’s instructions. The protein concentrations of the tissue homogenates were determined *via* bicinchoninic acid assay (Walker, 2009).

### Enzymatic Activities of the Mitochondrial Respiratory Chain Complex

The enzymatic activities of mitochondrial respiratory chain (MRC) complexes I and IV of the cells treated with ATR (0, 2.5, 5, 7.5, and 10 μM) for 24 h were determined with a microplate reader using commercially available kits (Solarbio, Beijing, China) in accordance with the manufacturer’s instruction. Briefly, mitochondrial homogenates were added into the respective reaction buffer. The reaction mixture was transferred to a prewarmed (30°C) quartz cuvette and immediately put into a spectrophotometer. The absorbance of reaction mixture was measured at 340 nm for Complex I, or 550 nm for Complex IV, respectively. Mitochondrial complex activity was expressed as nmol/min/mg protein. Three independent replicates were performed for each treatment.

### Measurement of Mitochondrial Membrane Potential

Mitochondrial membrane potential was detected using a mitochondrial membrane potential (MMP) assay kit with JC-1 (Beyotime Biotechnology, Jiangsu, China) in accordance with the manufacturer’s instruction. Briefly, the cells were washed three times with PBS and stained with 10 μg/ml JC-1 for 15 min at 37°C in the dark after treatment with FFA and ATR. Subsequently, the cells were rinsed with PBS three times and immediately observed by a laser scanning confocal microscope. The green fluorescence of J-monomer and the red fluorescence of J-aggregates were detected with 490/530 nm and 525/590 nm excitation/emission filters, respectively.

### Quantitative Real-Time PCR

The mRNA level of LC3B was determined with the SYBR Green II chimeric fluorescence method. Total RNA was extracted from cells by using the RNAiso Plus kit (TaKaRa Biotechnology, Dalian, China) in accordance with the manufacturer’s protocol. RNA integrity was checked *via* agarose gel electrophoresis with ethidium bromide staining. RNA concentration and purity were determined using an automatic microplate reader (Synergy H4, BioTek, Tokyo, Japan) at an OD 260/280 reading ratio of 1.8-2.1. After determining the RNA concentration, 1 μg of total RNA was reverse-transcribed into complementary DNA (cDNA) by using the PrimeScript RT reagent kit (TaKaRa Biotechnology). Real-time PCR was performed with an ABI StepOnePlus real-time PCR system (Applied Biosystems, Grand Island, NY) using the SYBR Premix Ex Taq II Reagent Kit (TaKaRa Biotechnology) in accordance with the manufacturer’s instructions but with modification (Zhang P. et al., 2018). The volume of the reaction system was 20 μl, including 10 μl of SYBR Premix Ex Taq П, 0.4 μl of PCR forward primer (10 µM), 0.4 μl of PCR reverse primer (10 µM), 2 μl of cDNA, and 7.2 μl of RNase-free H_2_O. The primer sequences of the target and reference genes (LC3B: Forward-GATGTCCGACTTATTCGAGAGC, Reverse-TTGAGCTGTAAGCGCCTTCTA; β-Actin: Forward-GATGGTGGGCATGGGTCAGAAGGA, Reverse-CATTGTAGAAGGTGTGGTGCCAGAT) were obtained from GenBank. The relative levels of mRNA expression were calculated using the 2^-ΔΔCT^ method after normalization against the reference gene β-actin (Livak and Schmittgen, 2001).

### Western Blot Analysis

All cell lysates were prepared by harvesting cells in RIPA lysis buffer (Beyotime Biotechnology, Jiangsu, China), followed by centrifugation at 12,000 rpm for 15 min at 4°C. Supernatants containing total proteins were quantified with a BCA protein estimation kit (Meilun Biotechnology, Dalian, China), and 50 μg of the total protein samples were resolved with 8%, 10%, or 15% polyacrylamide gel (depending on the molecular size of the proteins to be analyzed). Thereafter, immunoblotting was performed by transferring the resolved protein onto a PVDF membrane in a trans-buffer at 100 V for 1 or 2 h depending on the molecular size of the protein. Furthermore, the PVDF membranes were blocked with 5% skimmed milk or 5% BSA (for phosphorylated protein) in TBST buffer (20 mM Tris-HCl, pH 7.5, 150 mM sodium chloride, and 0.05% Tween 20) for 2 h, washed thrice with TBST buffer for 5 min each, and incubated overnight with primary antibodies (1:1000) at 4°C. Afterward, the membranes were washed thrice with TBST buffer for 5 min each time and incubated with anti-rabbit secondary antibodies conjugated to horseradish peroxidase (1:2000) for 2 h at room temperature. The blots were developed in the dark by using an ECL detection kit. The developed blots were subjected to densitometric analysis using Image J 1.45 software (NIH, USA) and normalized with β-actin as the internal control.

### Analysis of Autophagic Flux

For the determination of autophagic flux in response to ATR, HepG2 cells were transfected with a tandem fluorescent PCDH-CMV-mRFP1-EGFP-tagged LC3 lentivirus to obtain cells expressing mRFP-GFP-LC3. Thereafter, HepG2 cells expressing mRFP-GFP-LC3 were seeded on a six-well plate and treated with FFA and ATR. After incubation, autophagic flux was visualized using a laser scanning confocal microscope (LSM 800, Carl Zeiss, Germany). Images were acquired with Image J 1.45 software. Green puncta (GFP signal) represent early autophagosomes, and red puncta (mRFP signal) denote late autolysosomes. Autophagic flux was evaluated by the color change of GFP/mRFP.

### Colocation of LC3 Protein With Lipid Droplets

The cells were seeded on sterile coverslips at a density of 2.5 × 10^5^ cells/coverslip to study the colocation of LC3 protein with LDs. Then, FFA-induced steatosis was implemented, followed by ATR treatment for 24 h. After the completion of incubation, the cells were fixed with 4% formaldehyde for 15 min and washed with PBS thrice for 5 min each time. The fixed cells were permeabilized with 0.1% Triton X-100 for 10 min then washed and blocked with 2% BSA for 1 h at room temperature. After blocking, the cells were incubated with LC3B antibody (1:1000) overnight at 4°C, followed by incubation with the DyLight™ 680 conjugated secondary antibody (1:500) for 1 h at room temperature. Furthermore, the cells were washed and incubated with BODIPY (5 μg/ml in PBS) for 20 min at room temperature and counterstained with DAPI for 5 min to stain the nucleus. The coverslips were mounted on slides and visualized under a confocal microscope equipped with 405, 488, and 543 nm lasers (LSM 800, Carl Zeiss, Germany).

### Statistical Analysis

Quantitative data were presented as the mean ± SD of three independent experiments and statistically evaluated *via* one-way ANOVA followed by Tukey’s multiple comparison test using the Graph Pad Prism 6 software (GraphPad software, San Diego, CA, USA). In the data analysis, P<0.05 was considered statistically significant.

## Results

### Safe Concentrations of ATR and Level Changes of ADP/ATP

**Figure 1:**
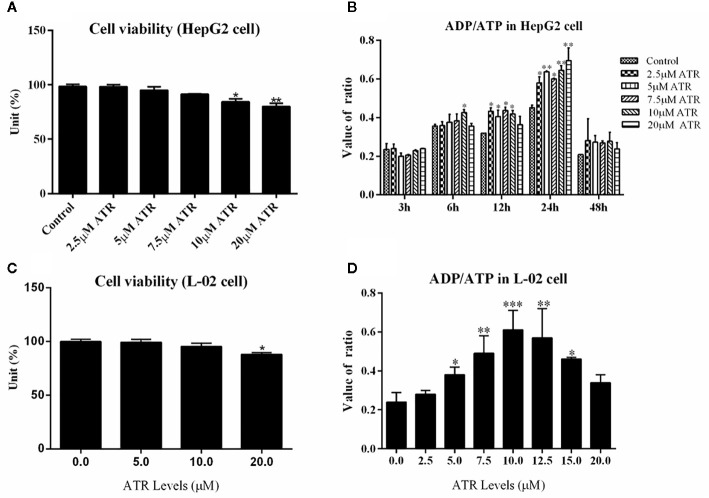
Safe concentration of ATR and upregulation of ADP/ATP in hepatocytes (HepG2 and L-02 cells). **(A)** Cell viability of HepG2 cells exposed to ATR (CCK-8 assay). **(B)** Value of ADP/ATP in ATR-treated HepG2 cells. **(C)** Cell viability of L-02 cells exposed to ATR (CCK-8 assay). **(D)** Value of ADP/ATP in ATR-treated L-02 cells (cells were treated with ATR for 24 h). Data are expressed as mean ± SD (n=3). *P<0.05, **P<0.01, ***P<0.001 (*represents ATR groups compared with the control group).

The viability of HepG2 cells and L-02 cells was measured *via* CCK-8 assay. ATR inhibited the viability of HepG2 cells at concentrations of 10 and 20 μM (**Figure 1A**), but low-concentration ATR (2.5, 5, and 7.5 μM) had no effect on the viability of HepG2 cells. The values of ADP/ATP in the cells increased significantly when the cells were treated with ATR for 12 and 24 h (**Figure 1B**). The largest increment in ADP/ATP was observed at 24 h with the inclusion of all ATR groups. The ratio of ADP/ATP decreased when the cells was treated with ATR for 48 h (compared with 3 h). The viability of L-02 cells was used to confirm the safe concentration of ATR further. We found that only 20 μM ATR inhibited cell viability (**Figure 1C**). And, the values of ADP/ATP increased significantly in the 5, 7.5, 10, 12.5, and 15 μM ATR groups (**Figure 1D**). This finding suggests that low-concentration ATR (lower than 10 μM) has no side effect and can increase the ratio of ADP/ATP in cells treated with ATR for 24 h. Additionally, The long-term effect of low concentration (0.5, 1, and 2 mg/kg) of ATR on mice was investigated after mice was intraperitoneal injected with ATR for 2 weeks. The results showed that body temperature was significantly increased (2–3°C higher than that of the control group; **Supplementary Figure 2C**), the body weight gain (**Supplementary Figure 2A**) and relative weight of epididymis fat (**Supplementary Figure 2B**) were decreased slightly. Besides, low-dose ATR had no obvious toxic and side effects on the liver and kidney of mice (**Supplementary Figures 2D–G**).

### ATR Accelerates Intracellular Lipid Degradation in FFA-Treated HepG2 Cells

**Figure 2:**
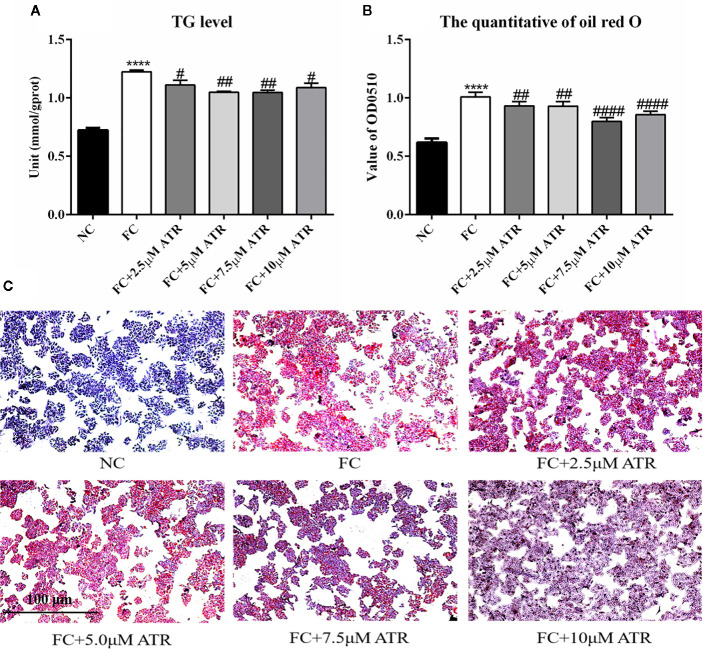
Effect of ATR on TG level in FFA-treated HepG2 cells. **(A)** Changes in TG level in FFA-treated HepG2 cells. **(B)** Quantity of oil red O in FFA-treated HepG2 cells. **(C)** Images (5×) of HepG2 cells stained with oil red O. Data are expressed as mean ± SD (n=3). ****P<0.001 (*represents ATR groups compared with the control group). **^#^**P<0.05, **^##^**P<0.01, **^####^**P<0.001 (**^#^**represents FC+ATR treatments compared with the FC group). NC=normal group, and FC=FFA group.

After identifying the safe concentrations of ATR, the effect of low-concentration ATR on FFA-induced lipid accumulation was measured. As shown in **Figure 2A**, the intracellular TG level of cells remarkably increased in the FFA control (FC) group compared with the normal control (NC) group, and the cell viability was not affected (**Supplementary Figure 1**). However, the cells co-treated with FFA and ATR exhibited reduced TG levels compared with the FC group (**Figure 2A**). The intracellular lipid was analyzed *via* oil red O staining (**Figure 2C**), and the results showed that lipid accumulation in the FFA-treated cells was reduced in the presence of ATR. The quantification of stored lipids showed that ATR treatment reduced the lipid content compared with the FC group, and the lowest lipid content was observed in the 7.5 μM ATR group (**Figure 2B**). Altogether, these results imply that ATR can accelerate the accumulated lipid degradation in FFA-treated HepG2 cells.

### Mitochondrial Adaptation of ATR in FFA-Treated HepG2 Cells

**Figure 3:**
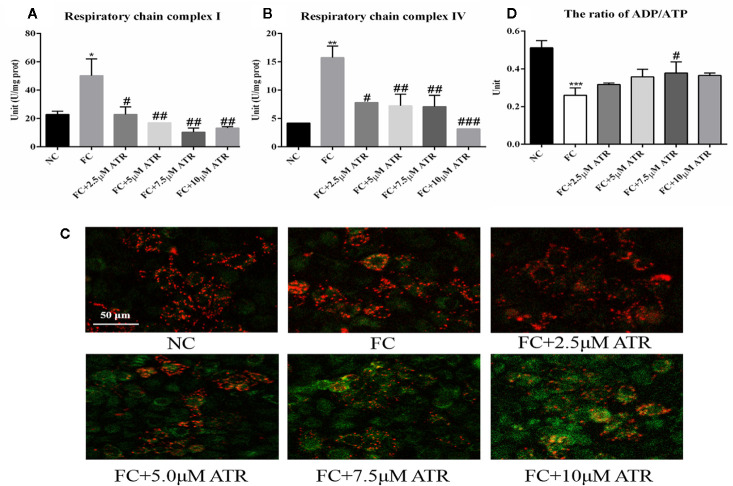
Mitochondrial adaptation effect of ATR on FFA-treated HepG2 cells. **(A)** Activity of mitochondrial respiratory chain complex I. **(B)** Activity of mitochondrial respiratory chain complex IV. **(C)** Changes in JC-1 fluorescence in response to ATR (red fluorescence represents normal mitochondrial membrane potential and green fluorescence represents decreased mitochondrial membrane potential). **(D)** Ratio of ADP/ATP. Data are expressed as mean ± SD (n=3). *P<0.05, **P<0.01, ***P<0.001 (*represents the FC group compared with the NC group). **^#^**P<0.05, **^##^**P<0.01, **^###^**P<0.001 (**^#^**represents FC+ATR treatments compared with the FC group). NC=normal group, and FC=FFA group.

The mitochondrial adaptation effect of ATR has been reported before, and it may lead to the inhibition of lipid accumulation in HepG2 cells. Thus, the mitochondrial adaptation effects of ATR were determined in the current study. The activity of MRC complexes I and IV significantly increased under FFA treatment (FC vs. NC, **Figures 3A, B**). However, the activity of respiratory chain complexes I and IV in the cells of the FC group significantly decreased with the inclusion of ATR at all doses (FC+ATR vs. FC, **Figures 3A, B**). The MMP was measured *via* JC-1 staining. ATR decreased the MMP of cells (**Figure 3D**). Furthermore, the cells treated with ATR increased the reduction ratio of ADP/ATP induced by FFA, but statistical significance was only found in the 7.5 μM ATR group (**Figure 3C**).

### AMPK/mTOR Signaling Pathway Induced by ATR

**Figure 4:**
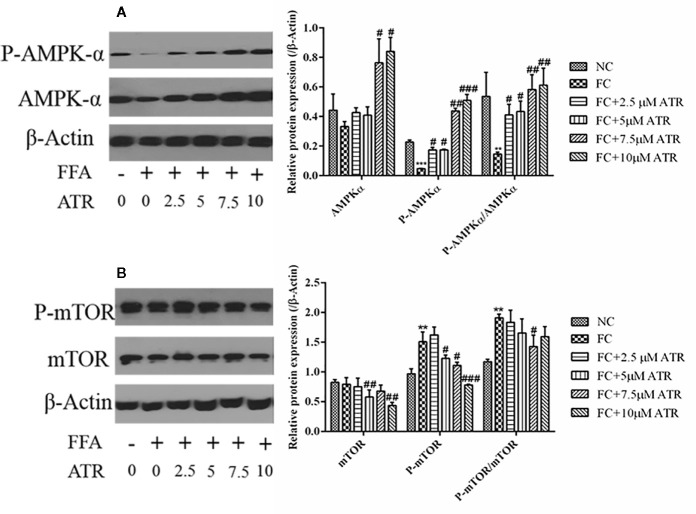
Effect of ATR on the protein expression of AMPKα and mTOR in FFA-treated HepG2 cells. **(A)** Immunoblot analysis for AMPK-α, phosphorylated AMPKα (p-AMPKα, Thr 172), and relative protein expression in reference to β-actin. **(B)** Immunoblot analysis for mTOR, phosphorylated mTOR (p-mTOR, Ser 2448), and relative protein expression in reference to β-actin. Data are expressed as mean ± SD (n=3). **P<0.01, ***P<0.001 (*represents the FC group compared with the NC group). ^#^P<0.05, ^##^P<0.01, ^###^P<0.001 (^#^represents FC+ATR treatments compared with the FC group). NC=normal group, and FC=FFA group.

As shown in **Figure 4**, FFA treatment decreased the protein expression of p-AMPKα (Thr 172) and the value of p-AMPKα/AMPKα; it also increased the protein expression of p-mTOR (Ser 2448) and the value of p-mTOR/mTOR. In contrast to the FC group, the 7.5 and 10 μM ATR groups had a significantly increased AMPKα protein level, but all doses of ATR increased the p-AMPKα (Thr 172) protein level and value of p-AMPKα/AMPKα (P<0.05; **Figure 4A**). With regard to the protein expression of mTOR, 5 and 10 μM ATR treatments significantly decreased the protein level of mTOR and p-mTOR (Ser 2448), but only the 7.5 μM ATR treatment significantly decreased the value of p-mTOR/mTOR.

### Autophagy Induced by ATR in FFA-HepG2 Cells

**Figure 5:**
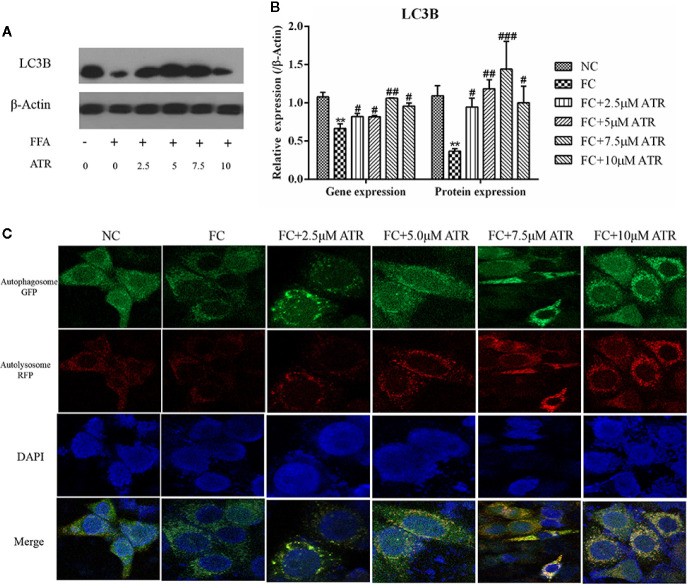
Gene and protein expression of LC3B and autophagic flux analysis. **(A)** Immunoblot analysis for LC3B in reference to β-actin. **(B)** Gene and protein expression of LC3B in reference to β-actin. **(C)** Representative confocal images of cells stably expressing mRFP-GFP-LC3. Scale bars: 10 μm. Data are expressed as mean ± SD (n=3). **P<0.01 (*represents the FC group compared with the NC group). ^#^<0.05, ^##^P<0.01, ^###^P<0.001 (**^#^**represents FC+ATR treatments compared with the FC group). NC=normal group, and FC=FFA group.

The LC3B expression and autophagic flux were determined to analyze the role of autophagy in ATR’s lipid inhibitory activity. As shown in **Figure 5**, FFA treatment decreased the gene and protein expression levels (FC vs. NC; **Figures 5A, B**). In contrast to the FC group, the FFA-treated cells with ATR increased the LC3B gene and protein levels (P<0.05), and the highest levels were observed at the dose of 7.5 μM. In addition, the autophagic flux results showed that ATR treatment increased the green (GFP signal) and red (mRFP signal) puncta, and the number of red puncta was greater than that of green puncta in the merged images (**Figure 5C**).

### ATR Promoted LC3 Protein Expression in LDs

BODIPY staining was used to visualize the changes of LDs in cells. As shown in **Supplementary Figure 3A**, ATR treatment reduced the stored LDs in FFA-treated cells (stained by BODIPY). The average optical density analysis confirmed that ATR treatment reduced the fluorescence intensity of LDs, and statistical significance was observed in the 7.5 and 10 μM ATR groups (**Supplementary Figure 3B**).

**Figure 6:**
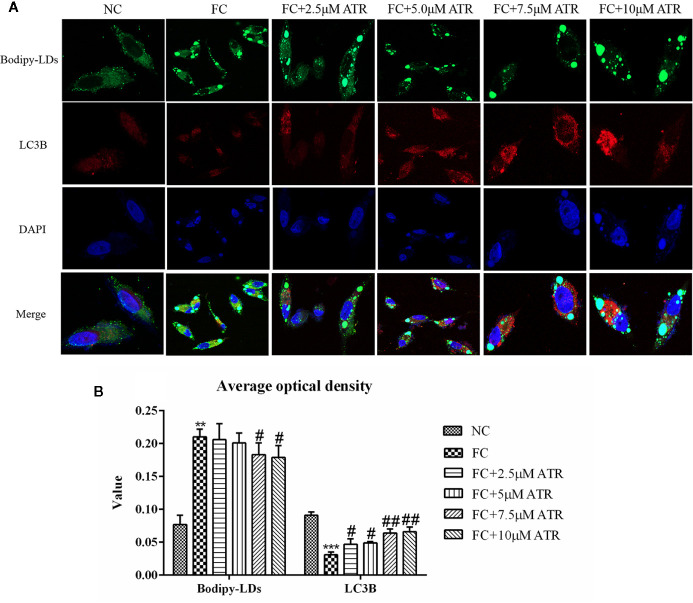
ATR treatment promoted LC3 protein expression in LDs. **(A)** Representative confocal images of co-localization of DyLight 680-stained LC3B protein with BODIPY-stained LDs. Scale bars: 10 μm. **(B)** Optical density analysis of fluorescence intensity. Data are expressed as mean ± SD (n=3). **P<0.01, ***P<0.001 (* represents the FC group compared with the NC group). ^#^P<0.05, ^##^P<0.01 (^#^represents FC+ATR treatments compared with the FC group). NC=normal group, and FC=FFA group.

Colocation of LC3B and LDs was performed to determine if the reduced LDs were associated with autophagy. As shown in **Figure 6A**, the cells treated with FFA exhibited a limited number of co-localizations of LC3B and LDs (**Figure 6A**, column 2), as evidenced by limited green and red puncta. Meanwhile, the cells co-treated with FFA and ATR showed an increased number of co-localizations of LC3B and LDs, as indicated by increased red and decreased green puncta. The optical density analysis showed that ATR treatment reduced the green fluorescence intensity of LDs and increased the red fluorescence intensity of LC3B (**Figure 6B**; P<0.05). These data reveal the direct association of LDs and LC3B in ATR-treated steatosis HepG2 cells.

## Discussion

ATR, a diterpenoid glycoside known for its specific oxidative phosphorylation inhibitory effect, is found in the fruits of *Xanthium Sibiricum* (Stewart and Steenkamp, 2000). Recent reports have shown that carboxy-atractyloside (cATR) specifically inhibits liver ANT2 and improves the liver steatosis level by reducing liver TG accumulation (Shabalina et al., 2006; Cho et al., 2017). This finding implies that ATR [same structure as cATR, except for the lack of an extra carboxylate group (Sanchez et al., 2012)] may have similar effects. In this study, we identified a novel mechanism of lipid mobilization in HepG2 cells mediated by ATR-induced autophagy.

High-concentration ATR is known to be toxic to the liver and kidney, but low concentrations have no side effect (Yu et al., 2013). We investigated the safe concentrations of ATR in HepG2 and L-02 cells. The results showed that low doses of ATR (2.5, 5, 7.5, and 10 μM) had no effect on the viability of HepG2 and L-02 cells. ATR treatment significantly increased the level of ADP/ATP in cells in a positive dose-dependent manner, and the highest level of ADP/ATP was observed in cells treated with ATR for 24 h. Meanwhile, the level of ADP/ATP decreased at 48 h because the accumulation of ADP reversed the effect of ATR (Stewart and Steenkamp, 2000). In addition, present study also proved the long-term effect of low concentration (0.5, 1, and 2 mg/kg) ATR had no obvious toxic and side effects on the liver and kidney of mice (**Supplementary Figure 2**). These results further prove the safety of low-concentration ATR. Thus, low concentrations of ATR (2.5, 5, 7.5, and 10 μM) were used for the subsequent HepG2 cells steatosis experiments, and the treatment time was 24 h.

Chronic exposure to lipids or acute exposure to high concentrations of lipids may change the lipid accumulation in hepatocytes. Similar to a previous study, the current study showed that FFA treatment significantly increased the intracellular TG level of HepG2 cells. However, cells co-treated with FFA and ATR exhibited a significant reduction in TG and LD levels. The lowest lipid content was observed in the 7.5 μM ATR treatment group. This finding suggests that ATR can accelerate the accumulated lipid degradation in FFA-treated HepG2 cells, which is similar to the effect of the *Frutus Xanthii* decoction that attenuates high fatty diet induced hepatic steatosis (Li et al., 2013).

The decreased level of TG and LDs induced by ATR may be related to the effect of ATR on energy balance (ADP/ATP level). Moreover, carnitine palmitoyl-transferase 1 (CPT1) mediates FFA entry into mitochondria, leads to reduced nicotinamide adenine dinucleotide (NADH), transfers electrons to MRC, increases the MRC complex activity, and facilitates ATP synthesis (Fromenty and Pessayre, 1995; Pessayre et al., 2012). Additionally, FFAs can increase the activity of ANT to promote ADP transfer into mitochondrial matrix for ATP production (Lee et al., 2014). Thus, a reduced ratio of ADP/ATP and increased MRC complexes I and IV induced by FFA were observed in the present study. A previous study reported that liver-specific ANT2 depletion (cATR as the inhibitor) has no effect on the hepatic ATP level (Cho et al., 2017). But we observed that ATR increased the level of ADP/ATP in FFA-treated cells, which may be due the comprised ADP/ATP exchange capacity (Cho et al., 2017), resulting the accumulation of ADP. Furthermore, the activity of MRC complexes I and IV and the mitochondrial membrane potential were significantly decreased by ATR treatment, indicating that ATR inhibited mitochondrial overheating. These results suggest that the mitochondrial energy adaptation induced by ATR is related to the inhibition of the activity of MRC complexes I and IV and the reduced mitochondrial membrane potential; this effect is similar to that of berberine (Sun et al., 2018).

AMPK is known as a key regulator of cellular lipid metabolism and a nutrient sensor that sustains cellular energy homeostasis (Hardie, 2018). AMPK activation regulates lipid metabolism, and its inhibition is associated with obesity in lipid-overload conditions (Ke et al., 2018). In this study, we found that ATR significantly increased the protein levels of AMPKα and p-AMPKα and increased the ratio of p-AMPKα/AMPKα, suggesting that ATR induced the AMPK signaling pathway. Meanwhile, a previous study strongly suggested that AMPK is a positive regulator of autophagy and acts by downregulating the phosphorylation of mTOR (Kim et al., 2011). ATR exerted a similar effect in the current study; it decreased the protein level of mTOR and P-mTOR, but only the 7.5 μM ATR group had a significantly decreased value of p-mTOR/mTOR. Altogether, these results suggest that the AMPK-mTOR signaling pathway is involved in ATR-mediated lipid clearance of HepG2 cells.

The key role of autophagy in hepatic lipid degradation in various cell types, including adipocytes and hepatocytes, and in dysfunctional autophagy might lead to the accumulation of lipids and TGs (Kwanten et al., 2016). The upregulation of AMPK and downregulation of mTOR observed in the present study may have induced autophagy. The gene and protein expression levels of LC3B were increased by ATR treatment. In addition, ATR promoted the autophagic flux processed from early autophagosome to late autolysosome, as evidenced by the increased red puncta (mRFP signal) in the confocal images of cells stably expressing mRFP-GFP-LC3. These data reveal that autophagy may be involved in ATR-mediated reduction of stored lipids.

Subsequently, co-localization of LDs with autophagosome was determined to confirm the link between ATR-induced autophagy and decreased lipid content. Cells co-treated with FFA and ATR showed increased LC3B and reduced LDs, as indicated by the increased red and decreased green puncta. However, few co-localizations were observed. The small number of LDs and few co-localizations with LC3B in the presence of ATR could be due to the rapid degradation of LDs. Therefore, ATR-mediated lipophagy was involved in the degradation of FFA-induced LDs in HepG2 cells.

In summary, the results of the present study demonstrated that low concentrations (2.5, 5, and 7.5 μM) of ATR treatment could activate autophagy to accelerate the degradation of TGs in steatosis HepG2 cells; the mechanism may be related to the activation of the AMPK/mTOR pathway induced by the increased ADP/ATP ratio. In addition, the ideal concentration of ATR for improving steatosis level of HepG2 cells was 7.5 μM. The current study provides a theoretical basis for ATR-induced autophagy for the prevention and treatment of FLD.

## Data Availability Statement

The raw data supporting the conclusions of this article will be made available by the authors, without undue reservation.

## Author Contributions

PZ participated in the literature search, study design, surgery operation, data collection, data analysis, data interpretation, and writing of the manuscript. CZ and TZ carried out the data analysis and provided the critical revision of the manuscript. LL, HS, YZ, and GZ conceived the study and participated in its design and coordination. All authors contributed to the article and approved the submitted version.

## Funding

This present study was supported in part by the National Natural Science Foundation of China (grant numbers: 81660755), the Science and Technology Project of Shenzhen of China (grant numbers: JCYJ20170307160524377 and JCYJ20190808162605484), and the Science and Technology Project of Longhua District of Shenzen (grant numbers: 2017083).

## Conflict of Interest

The authors declare that the research was conducted in the absence of any commercial or financial relationships that could be construed as a potential conflict of interest.

## Supplementary Material

Supplementary Figure 1 The viability of HepG2 cells treated with FFA. Cell viability was analyzed by CCK-8 assay. Data are expressed as mean ± SD (n=3).

Supplementary Figure 2 Changes of basic metabolism of mice treated with low-concentration ATR. 16 male C57BL/6 mice were (4 mice/4 treatments) intraperitoneal injected with different concentrations (0.5, 1, and 2 mg/kg) of ATR for 2 weeks. **(A)** Body weight gain of mice in 14 days. **(B)** Relative weight of liver, kidney, and Epididymal fat of mice. **(C)** Body Temperature changes of mice in 14 days. **(D) (E) (F)** The level of alanine aminotransferase (ALT), aspartate aminotransferase (AST), and triglycerides (TG) in serum and liver homogenate. **(G)** H&E staining of liver and kidney slices. Data are expressed as mean ± SD (n=4). *P<0.05, **P<0.01 (* represents ATR groups compared with the control group).

Supplementary Figure 3 BODIPY-stained LD level in HepG2 cells. **(A)** Representative confocal images of LDs stained by BODIPY and counterstained by DAPI. Scale bars: 50 μm. **(B)** Optical density analysis of fluorescence intensity. Data are expressed as mean ± SD (n=3). **P<0.01 (* represents the FC group compared with the NC group). ^#^P<0.05 (**^#^**represents FC+ATR treatments compared with the FC group). NC=normal group, and FC=FFA group.
