# Supplementary figures and images for: Mitochondrial Energy-Regulating Effect of Atractyloside Inhibits Hepatocellular Steatosis Through the Activation of Autophagy
Source: Front Pharmacol. 2020 Sep 30;11:575695. doi: 10.3389/fphar.2020.575695 (PMC7556285; doi:10.3389/fphar.2020.575695)

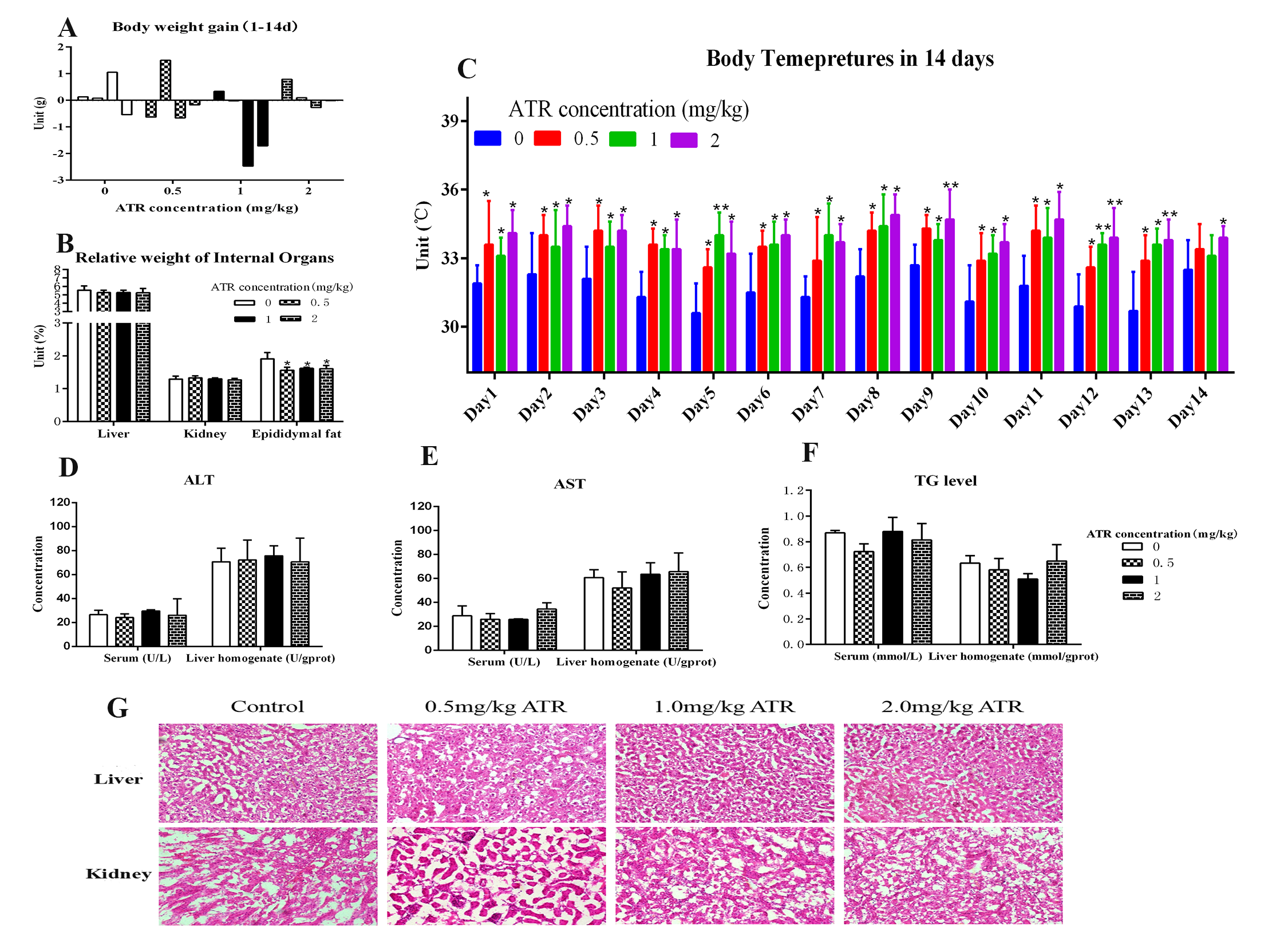

Supplement: Supplementary Figure 2 — Changes of basic metabolism of mice treated with low-concentration ATR. 16 male C57BL/6 mice were (4 mice/4 treatments) intraperitoneal injected with different concentrations (0.5, 1, and 2 mg/kg) of ATR for 2 weeks. (A) Body weight gain of mice in 14 days. (B) Relative weight of liver, kidney, and Epididymal fat of mice. (C) Body Temperature changes of mice in 14 days. (D) (E) (F) The level of alanine aminotransferase (ALT), aspartate aminotransferase (AST), and triglycerides (TG) in serum and liver homogenate. (G) H&E staining of liver and kidney slices. Data are expressed as mean ± SD (n=4). *P<0.05, **P<0.01 (* represents ATR groups compared with the control group). [file Image_2.tif]

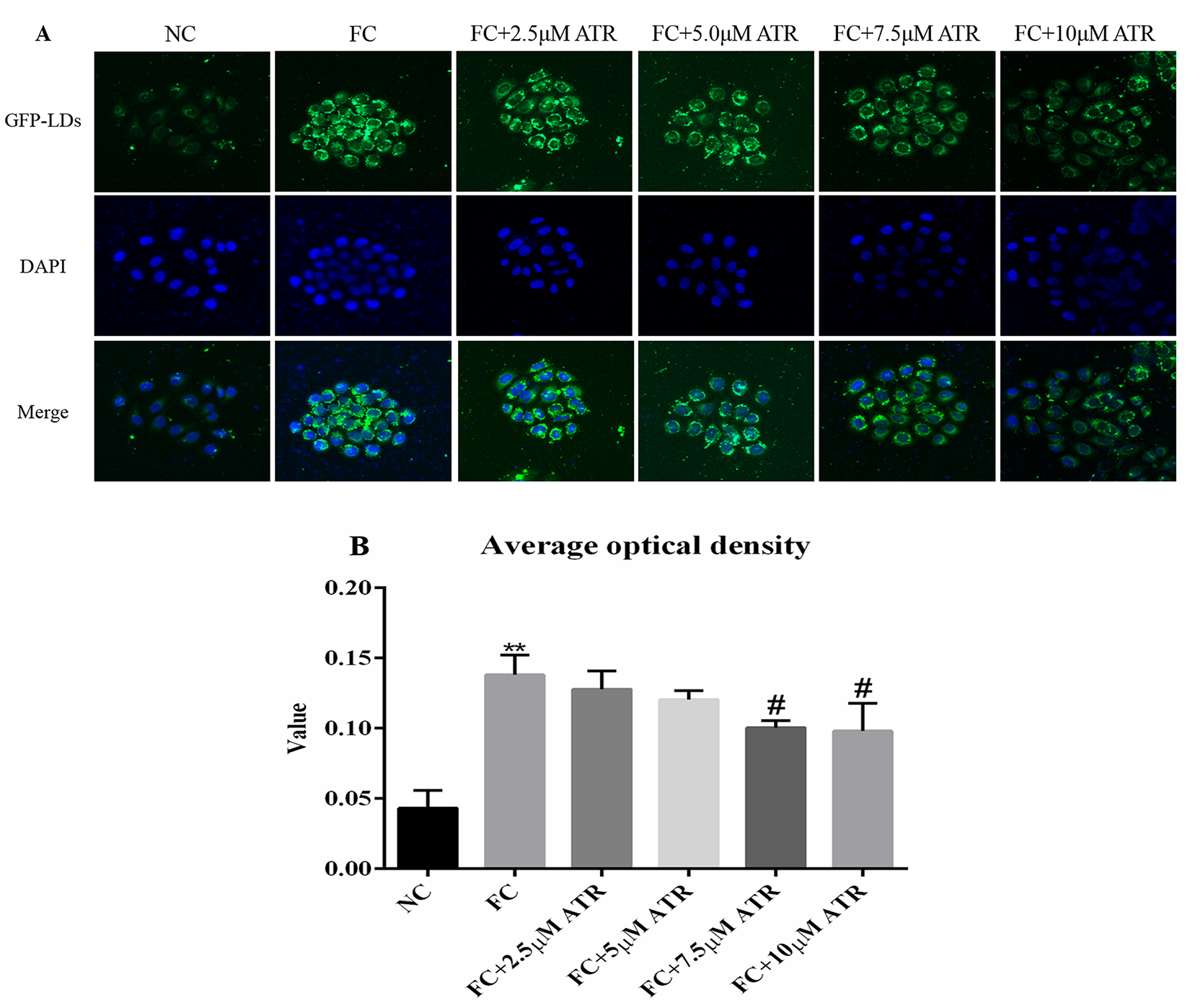

Supplement: Supplementary Figure 3 — BODIPY-stained LD level in HepG2 cells. (A) Representative confocal images of LDs stained by BODIPY and counterstained by DAPI. Scale bars: 50 μm. (B) Optical density analysis of fluorescence intensity. Data are expressed as mean ± SD (n=3). **P<0.01 (* represents the FC group compared with the NC group). #P<0.05 (#represents FC+ATR treatments compared with the FC group). NC=normal group, and FC=FFA group. [file Image_3.tif]
